# Polyarteritis nodosa presenting as sixth nerve palsy: a case report

**DOI:** 10.3389/fneur.2026.1793313

**Published:** 2026-05-04

**Authors:** João Mendes, Francisco Mendes, Ana Filipa Salvado, Diogo Valente Fortunato, João Vasco Garrido, Rita Condesso, Augusto Candeias

**Affiliations:** 1 Hospital do Espírito Santo de Évora, Évora, Portugal; 2 Cullen Eye Institute, Baylor College of Medicine, Houston, TX, United States

**Keywords:** abducens nerve palsy, cranial neuropathy, orbital apex syndrome, polyarteritis nodosa, vasculitic neuropathy

## Abstract

**Introduction:**

Polyarteritis nodosa (PAN) is a rare systemic necrotizing vasculitis affecting medium-sized vessels. While peripheral neuropathy occurs in 50–75% of patients, cranial nerve involvement is exceptionally rare, affecting fewer than 2% of patients. We report a case of PAN presenting with isolated sixth nerve palsy and orbital apex syndrome.

**Case presentation:**

A 70-year-old man presented with the acute onset of painful left abducens nerve palsy. His medical history included 3 months of constitutional symptoms, polyarthralgia, unintentional weight loss, and progressive hearing loss. Orbital MRI revealed enhancement extending from the left orbital apex through the superior orbital fissure to the cavernous sinus. During corticosteroid tapering, he developed painful cutaneous nodules, abdominal pain, and sensorimotor polyneuropathy with foot drop, fulfilling the diagnostic criteria for PAN. Treatment escalation with high-dose corticosteroids resulted in clinical improvement, with complete resolution of diplopia and stabilization of systemic manifestations at 6-month follow-up.

**Conclusion:**

This case highlights PAN as a rare but important cause of painful cranial neuropathy with orbital apex involvement. Clinicians should maintain a high index of suspicion for systemic vasculitis in patients presenting with painful ophthalmoplegia accompanied by constitutional symptoms, peripheral neuropathy, or cutaneous manifestations. Early recognition and prompt immunosuppressive therapy are essential to prevent irreversible neurologic damage and life-threatening complications.

## Introduction

Polyarteritis nodosa (PAN) is a systemic necrotizing vasculitis predominantly affecting medium-sized arteries, with an estimated prevalence of 3.1 per million adults. The disease typically presents in middle-aged adults with a slight male predominance. Most cases are idiopathic, though historically up to 10% were associated with hepatitis B virus (HBV) infection, an association that has declined dramatically with widespread HBV vaccination.

The clinical presentation of PAN is heterogeneous, reflecting its multisystem involvement. Constitutional symptoms, including fever, fatigue, and weight loss, occur in 70–85% of patients. Peripheral neuropathy, typically presenting as mononeuritis multiplex or polyneuropathy, is one of the most frequent and early manifestations, affecting 50–75% of patients. Cutaneous involvement occurs in approximately 60–70% of cases and includes livedo reticularis, subcutaneous nodules, and skin necrosis. Gastrointestinal manifestations, present in approximately 50% of patients, range from abdominal pain to life-threatening mesenteric ischemia.

In contrast to peripheral neuropathy, cranial nerve involvement in PAN is exceptionally rare, affecting fewer than 2% of patients. When present, cranial neuropathies most commonly involve the oculomotor, trochlear, abducens, facial, and vestibulocochlear nerves (1–3). The mechanism is presumed to be ischemic injury to the cranial nerves via vasculitis of the vasa nervorum or, less commonly, ischemia of extraocular muscles or brainstem nuclei.

Ophthalmic manifestations occur in 10–20% of PAN patients and include retinal vasculitis, ischemic optic neuropathy, scleritis, and peripheral ulcerative keratitis. Orbital apex syndrome, characterized by ophthalmoplegia with involvement of cranial nerves II, III, IV, and VI, represents an extremely rare presentation of PAN.

The diagnosis of PAN is based on a combination of laboratory, angiographic, and clinical findings. Unlike anti-neutrophil cytoplasmic antibodies (ANCA)-associated vasculitis, the diagnosis is not typically made on serology but requires at least three of the 10 criteria proposed by the American College of Rheumatology (ACR): (1) weight loss of >4 kg; (2) livedo reticularis; (3) testicular pain from testicular artery ischemia; (4) myalgias, weakness, or leg tenderness; (5) mononeuropathy or polyneuropathy; (6) diastolic blood pressure >90 mm Hg; (7) elevated blood urea nitrogen >40 mg/dL or creatinine >1.5 mg/dL; (8) HBV infection; (9) arteriographic abnormalities showing visceral artery aneurysms or occlusions; and (10) polymorphonuclear neutrophils in small- or medium-sized artery biopsies.

Treatment of non-HBV-associated PAN depends on disease severity. For severe disease with life-threatening or organ-threatening manifestations, the 2021 ACR/Vasculitis Foundation guidelines conditionally recommend cyclophosphamide combined with high-dose glucocorticoids. For non-severe disease, glucocorticoids combined with steroid-sparing immunosuppressive agents such as azathioprine or methotrexate are recommended.

We present a case of PAN with the exceptionally rare initial manifestation of isolated sixth nerve palsy with orbital apex syndrome, contributing to the limited literature on cranial nerve involvement in this disease. To our knowledge, only one other case of PAN presenting as sixth nerve palsy has been recently reported in the medical literature (4).

## Case report

A 70-year-old man of European descent presented to the ophthalmology department with a 3-day history of left eye pain and horizontal diplopia.

His medical history was significant for type 2 diabetes mellitus managed with metformin. For the preceding 3 months, he had been under evaluation by internal medicine for progressive constitutional symptoms, including fatigue, polyarthralgia affecting the fingers and ankles, and unintentional weight loss of 5 kg. He also reported progressive left-sided hearing loss and had experienced deep vein thrombosis involving the internal and external saphenous veins 6 months prior.

Two weeks before presentation, he experienced an episode of left eye pain that resolved spontaneously within 1 week. The pain worsened with ocular movements. He also reported a similar episode of right eye pain 2 months earlier.

On neuro-ophthalmic examination, best-corrected visual acuity was 20/25 in both eyes. Pupils were equal and reactive, with no relative afferent pupillary defect. Ishihara color testing showed 7/13 correct plates bilaterally. Intraocular pressures were normal. Ocular motility examination demonstrated marked limitation of abduction, supralevoduction, and infralevoduction of the left eye, consistent with left abducens nerve palsy. Fundoscopic examination was unremarkable, with no evidence of optic disc edema or retinal vasculitis.

Automated visual field testing (Humphrey 30–2, SITA Standard) demonstrated moderate bilateral visual field loss, with a mean deviation (MD) of −6.01 dB in the right eye and −8.06 dB in the left eye.

The patient was admitted with a diagnosis of painful sixth cranial nerve palsy and initiated intravenous methylprednisolone 1 g daily for 3 days, followed by oral prednisone 1 mg/kg/day.

Extensive laboratory evaluation was performed to identify the underlying etiology. Complete blood count revealed mild normocytic anemia. Erythrocyte sedimentation rate was markedly elevated at 67 mm/h (reference range 0–20 mm/h). C-reactive protein was elevated at 42 mg/L (reference range <10 mg/L). Autoimmune serology, including antinuclear antibodies, ANCAs, complement levels (C3, C4), angiotensin-converting enzyme, lysozyme, anti-SSA, anti-SSB, and HLA-B27, was negative. Notably, anti-cyclic citrullinated peptide (anti-CCP) antibodies and anti-double-stranded DNA (anti-dsDNA) antibodies were positive at low titers, though the patient did not meet diagnostic criteria for rheumatoid arthritis or systemic lupus erythematosus.

Infectious disease workup, including serologies for syphilis (RPR, FTA-ABS), tuberculosis (QuantiFERON-Gold), Lyme disease, and Bartonella, was negative. Hepatitis B surface antigen, hepatitis B core antibody, and hepatitis C antibody were negative.

Hypercoagulability evaluation, including factor V leiden, prothrombin gene mutation, antiphospholipid antibodies, lupus anticoagulant, protein C and S activity, antithrombin III, β2-glycoprotein I antibody, anticardiolipin antibody, and cryoglobulins, was negative.

Paraneoplastic antibody panel and serum protein electrophoresis were negative. Cerebrospinal fluid analysis showed normal cell count, protein, and glucose with a normal CD4/CD8 ratio.

Computed tomography of the chest revealed bronchiectasis in the right lower lobe and ground-glass opacity in the superior segment. Positron emission tomography showed no hypermetabolic activity suggesting malignancy. MRI of the ears was normal.

**Figure 1:**
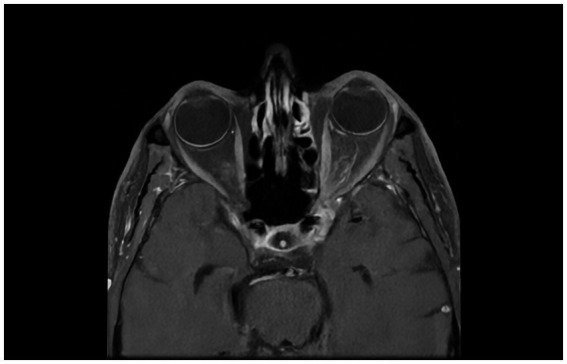
Axial T1-weighted, fat-suppressed, post-contrast MRI of the brain and orbits show enhancement in the left orbital apex involving the optic nerve sheath, without well-defined nodularity. The enhancement extends to the superior orbital fissure, the anterior aspect of the cavernous sinus, the pterygopalatine fossa, and the inferior orbital fissure.

Brain and orbital MRI with gadolinium contrast demonstrated abnormal enhancement in the left orbital apex involving the optic nerve sheath, extending posteriorly through the superior orbital fissure into the anterior cavernous sinus. Enhancement also extended inferiorly involving the pterygopalatine fossa and inferior orbital fissure, consistent with orbital apex syndrome (Figure 1).

The patient initially responded to intravenous methylprednisolone with improvement in eye pain. He was transitioned to oral prednisone with a planned taper. However, during corticosteroid tapering over the subsequent 4 weeks, he developed new manifestations, including painful, erythematous subcutaneous nodules on both forearms consistent with erythema nodosum, severe abdominal pain without a clear gastrointestinal source on imaging, progressive sensorimotor polyneuropathy with left foot drop, and worsening constitutional symptoms with increased fatigue.

Electromyography and nerve conduction studies confirmed sensorimotor polyneuropathy with axonal features consistent with mononeuritis multiplex.

Given the constellation of findings, including weight loss exceeding 4 kg, polyneuropathy, cutaneous nodules, and gastrointestinal symptoms, the patient fulfilled diagnostic criteria for PAN. The diagnosis was further supported by the absence of ANCA antibodies, absence of glomerulonephritis, and the pattern of medium-vessel involvement. The patient was readmitted and treated with high-dose intravenous methylprednisolone 1 g daily for 3 days, followed by oral prednisone 60 mg daily. With corticosteroid escalation, he experienced significant improvement in abdominal pain, stabilization of his neuropathy, and gradual improvement in diplopia over several weeks.

At 6-month follow-up, the patient demonstrated complete resolution of diplopia. His foot drop improved with physical therapy, though mild residual weakness persisted. Cutaneous nodules resolved completely. No new systemic manifestations developed. The patient was maintained on prednisolone 5 mg daily and methotrexate 10 mg weekly with folic acid supplementation.

## Discussion

This case illustrates an exceptionally rare presentation of PAN, with isolated cranial neuropathy as the initial manifestation. The patient ultimately fulfilled multiple diagnostic criteria for PAN, including constitutional symptoms with significant weight loss, polyneuropathy, cutaneous involvement, and gastrointestinal manifestations.

The initial presentation of painful ophthalmoplegia and orbital apex syndrome preceded the development of more classical systemic features by several weeks. This highlights the diagnostic challenge when cranial neuropathy presents as an early or isolated manifestation of systemic vasculitis. The painful nature of ophthalmoplegia was an important clinical clue, suggesting an inflammatory or ischemic etiology rather than isolated microvascular cranial neuropathy.

The MRI findings of enhancement extending from the orbital apex through the superior orbital fissure to the cavernous sinus represent an unusual manifestation of PAN. While ophthalmic involvement occurs in 10–20% of PAN patients, it typically manifests as retinal vasculitis, scleritis, or ischemic optic neuropathy rather than cranial neuropathy with orbital apex involvement. The mechanism is presumed to be vasculitis affecting the vasa nervorum of the sixth cranial nerve, potentially with extension to involve other structures within the orbital apex and cavernous sinus.

The presence of low-titer anti-cyclic citrullinated peptide (anti-CCP) and anti-double-stranded DNA (anti-dsDNA) antibodies was unexpected, as these are not typical serologic features of PAN. However, the patient did not fulfill classification criteria for rheumatoid arthritis or systemic lupus erythematosus, and there were no clinical or laboratory findings suggesting a connective tissue disease overlap. Although anti-CCP antibodies have been described in some patients with ANCA-associated vasculitis, their significance in PAN remains uncertain.

In this context, these low-titer autoantibodies were interpreted as non-specific findings, likely reflecting immune activation in the setting of systemic inflammation rather than a true overlap syndrome.

The differential diagnosis for painful ophthalmoplegia with orbital apex involvement is broad and includes infectious etiologies (fungal sinusitis, tuberculosis, syphilis), inflammatory conditions (sarcoidosis, IgG4-related disease, Tolosa–Hunt syndrome), neoplastic processes (lymphoma, metastases, perineural spread), and other vasculitides (giant cell arteritis, granulomatosis with polyangiitis) (Table 1).

**Table 1:** Differential diagnosis of painful ophthalmoplegia with orbital apex involvement.

Differential diagnosis
Polyarteritis nodosa
Granulomatosis with Polyangiitis (GPA)
Eosinophilic granulomatosis with polyangiitis
Microscopic polyangiitis
Systemic Lupus Erythematosus (SLE)
Relapsing polychondritis
Giant cell arteritis
Thyroid eye disease
Orbital myositis
IgG4
Tolosa-Hunt
Tuberculosis
Sarcoidosis
Syphilis
Lymphoma

Tolosa–Hunt syndrome was considered less likely for several reasons. While Tolosa–Hunt syndrome classically presents with painful ophthalmoplegia and granulomatous inflammation on MRI, it is fundamentally a diagnosis of exclusion. The patient’s constellation of systemic features, including constitutional symptoms, progressive hearing loss, weight loss, and subsequent development of peripheral neuropathy and cutaneous nodules, pointed toward a systemic inflammatory process rather than isolated idiopathic granulomatous inflammation of the cavernous sinus.

Giant cell arteritis (GCA) was initially considered, given the patient’s age (70 years), elevated inflammatory markers (erythrocyte sedimentation rate, 67 mm/h), and painful cranial neuropathy. However, several features made GCA less likely, including the absence of temporal artery abnormalities, the absence of jaw claudication, the absence of visual symptoms suggestive of ischemic optic neuropathy, and most importantly, the presence of peripheral neuropathy with mononeuritis multiplex and cutaneous nodules, manifestations not typical of GCA but characteristic of medium-vessel vasculitis such as PAN.

Although tissue diagnosis through biopsy remains the gold standard for confirming vasculitis, it was not pursued in this case. The patient fulfilled multiple ACR classification criteria for PAN, including weight loss >4 kg, polyneuropathy, cutaneous nodules, and gastrointestinal manifestations, supporting a robust clinical diagnosis. In addition, the overall clinical course, characteristic imaging findings, ANCA negativity, and clear response to immunosuppressive therapy further support the diagnosis. Deep skin biopsy of the subcutaneous nodules or a combined nerve–muscle biopsy could have provided histologic confirmation of necrotizing vasculitis affecting medium-sized vessels.

Cyclophosphamide combined with high-dose glucocorticoids is conditionally recommended for severe PAN with life-threatening or organ-threatening manifestations. This patient had several features suggesting severe disease, including progressive sensorimotor polyneuropathy with associated foot drop, severe abdominal pain, and cranial neuropathy with orbital apex syndrome.

However, the decision to use high-dose corticosteroids alone was based on several considerations: rapid and sustained clinical response to corticosteroid escalation, with improvement in abdominal pain, stabilization of neuropathy, and gradual resolution of diplopia; absence of immediately life-threatening complications, such as mesenteric infarction or bowel perforation on imaging; and cyclophosphamide toxicity concerns (bone marrow suppression, hemorrhagic cystitis, malignancy risk) in a 70-year-old patient with diabetes mellitus.

This case contributes to the limited literature on cranial nerve involvement in PAN. A systematic review of neurologic manifestations in PAN found that cranial neuropathies affect fewer than 2% of patients and typically occur later in the disease course rather than as presenting features. The sixth nerve is among the cranial nerves that can be affected, though isolated sixth nerve palsy as the initial manifestation is extraordinarily rare, with only one other recent case report in the literature.

## Conclusion

This case demonstrates that PAN should be considered in the differential diagnosis of painful cranial neuropathy, particularly when accompanied by constitutional symptoms, peripheral neuropathy, or cutaneous manifestations. Painful ophthalmoplegia should prompt consideration of inflammatory or vasculitic etiologies, not just microvascular ischemia. Cranial neuropathy may be an early or presenting feature of PAN, preceding more classic systemic manifestations. Early recognition and prompt immunosuppressive therapy are essential to prevent irreversible neurologic damage and life-threatening complications. While histopathological confirmation is ideal, a clinical diagnosis based on ACR criteria and therapeutic response is appropriate when biopsy is not feasible or would delay critical treatment.

The CARE Checklist has been completed by the authors for this case report and is attached as Supplementary material.

## Data Availability

The datasets presented in this article are not readily available because of ethical and privacy restrictions. Requests to access the datasets should be directed to the corresponding author.
